# Case Report: Four Cases of Cortical/Brainstem Encephalitis Positive for Myelin Oligodendrocyte Glycoprotein Immunoglobulin G

**DOI:** 10.3389/fneur.2021.775181

**Published:** 2022-01-21

**Authors:** Wan Wang, Juntao Yin, Zhiliang Fan, Juxian Kang, Jia Wei, Xiaoqian Yin, Shaohua Yin

**Affiliations:** ^1^Department of Neurology, Affiliated Hospital Xingtai People's Hospital, Hebei Medical University, Xingtai, China; ^2^Department of Neurology, Xingtai Third Hospital, Xingtai, China; ^3^Department of Imaging, Affiliated Hospital Xingtai People's Hospital, Hebei Medical University, Xingtai, China

**Keywords:** brainstem encephalitis, cortical encephalitis, epileptic seizures, myelin oligodendrocyte glycoprotein, neuromyelitis optica spectrum disorder

## Abstract

**Aim:**

Despite a significant improvement in the number of studies on myelin oligodendrocyte glycoprotein (MOG)-immunoglobulin G (IgG)-associated disorder (MOGAD) over the past few years, MOG-IgG-associated cortical/brainstem encephalitis remains a relatively uncommon and less-reported presentation among the MOGAD spectrum. This study aimed to report the clinical course, imaging features, and therapeutic response of MOG-IgG-associated cortical/brainstem encephalitis.

**Methods:**

Data of four patients who suffered from cortical encephalitis with epileptic seizures and/or brainstem encephalitis during the course of the disease were retrospectively collected and analyzed.

**Results:**

In this study, three male patients and one female patient, with a median age of onset of 21 years (ranging 20–51 years) were enrolled. An epileptic seizure was the main symptom of cortical encephalitis in these patients, while the manifestations of brainstem encephalitis were diverse. Cranial MRI demonstrated abnormal signals in unilateral or bilateral cortical or brainstem. Cerebrospinal fluid studies showed normal or mildly elevated leukocyte counts and protein levels, and a cell-based assay detected positive MOG-IgG in the serum of all patients. Two patients were misdiagnosed at the first attack, and both experienced a relapse. All of them accepted the first-line immunotherapy after a confirmed diagnosis and had a good outcome.

**Conclusion:**

Early suspicion of MOG-IgG-associated encephalitis is necessary for any patient with sudden onset of seizures or symptoms of brainstem damage, especially with lesions on unilateral/bilateral cortical or brainstem on brain MRI.

## Introduction

Myelin oligodendrocyte glycoprotein (MOG) is a myelin protein expressed on the outer surface of myelin sheaths and oligodendrocyte processes in the central nervous system (CNS) (1). Although MOG-immunoglobulin G (IgG)-positive cases account for about 25% of aquaporin-4 (AQP4)-seronegative neuromyelitis optica spectrum disorders (2), the clinical manifestation is less well-defined. In recent years, an enormous amount of research has been conducted to determine the role of MOG-IgG in a wide clinical spectrum of inflammatory demyelinating CNS disorders, including optic neuritis (ON), myelitis, cortical damage (3), and less commonly, brainstem lesions (4). Compared with ON and myelitis, cortical encephalitis with seizures and brainstem encephalitis are emerging presentations of MOG-IgG-associated disorder (MOGAD), and the related research has been rarely reported. The relationship between MOG-IgG and cortical encephalitis presentation was not recognized until the first case reported in 2017 (5). Subsequent case series further confirmed the association between MOG-IgG, fluid-attenuated inversion recovery (FLAIR)-hyperintense cerebral cortical lesions, and sudden epilepsy. Furthermore, most patients with MOG-IgG-associated cortical encephalitis had a relapsing disease course and experienced other demyelinating events, such as ON, myelitis, or brainstem damage, at a certain stage of the disease (6–10).

In the present study, four cases of MOG-IgG-associated cortical/brainstem encephalitis are reported, providing detailed information on the clinical manifestations, imaging features, disease evolution, and treatment outcomes of this disease.

## Case Presentation

### Case 1

**Figure 1:**
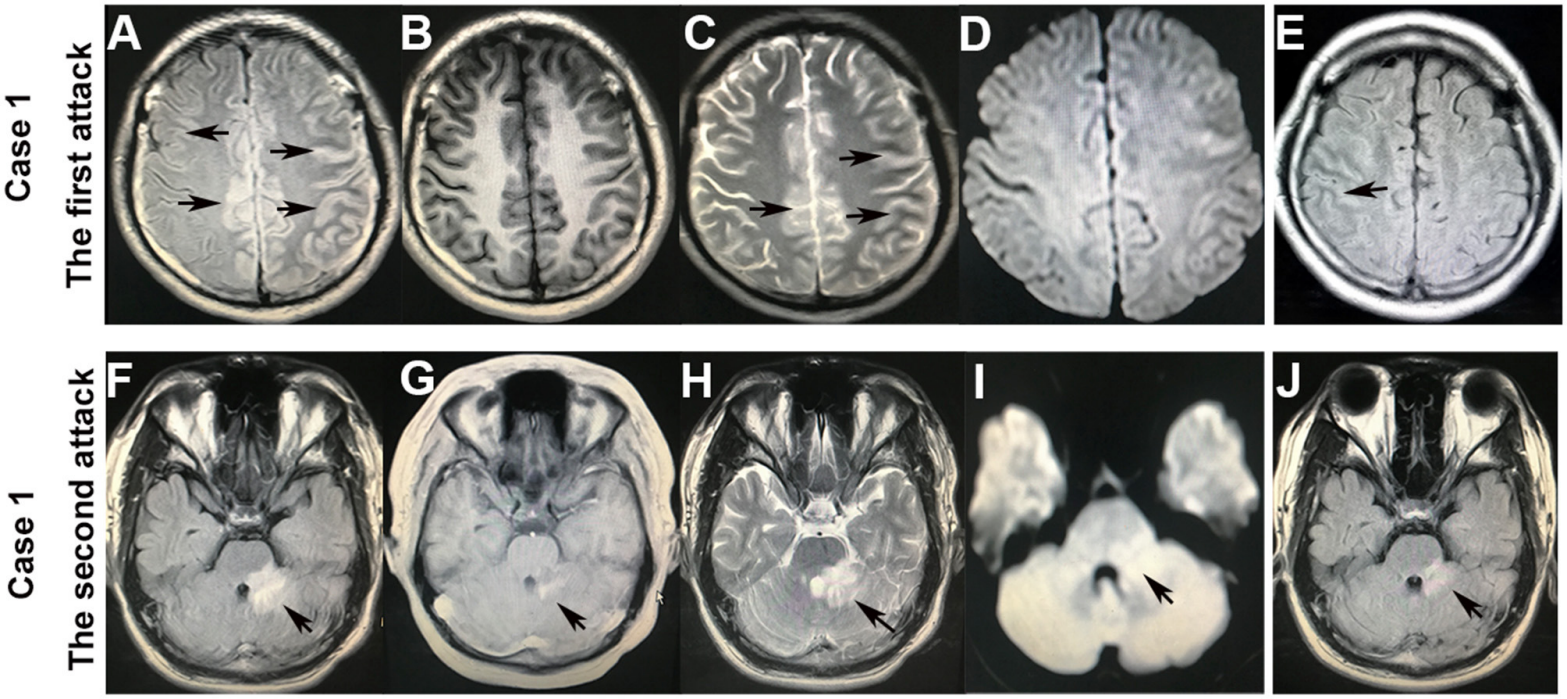
Brain MRI of case 1. After the first attack of an epileptic seizure, the hyperintense lesions with a hazy border in the bilateral cortex on fluid-attenuated inversion recovery (FLAIR) **(A)** and T2-weighted **(C)** images (arrowheads), which were less evident on T1-weighted **(B)** and diffusion-weighted **(D)** images. FLAIR hyperintensities in the cortical regions became shallow and fewer after 3 months **(E)** (arrowheads). After the second attack that presented with dizziness and left-sided ataxia, the hyperintensities in the pons and left brachium pontis on FLAIR **(F)**, T1-weighted **(G)**, T2-weighted **(H)**, and diffusion-weighted **(I)** images (arrowheads). The FLAIR hyperintensities in the brainstem became shallow and smaller after 1 month **(J)**.

**Figure 2:**
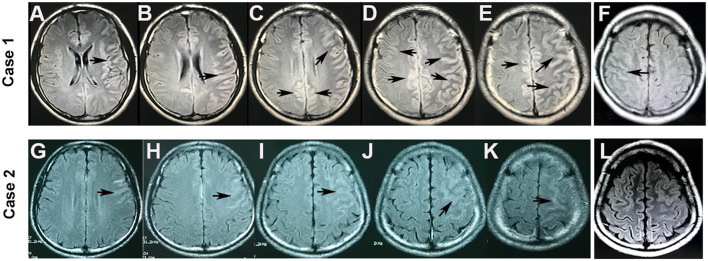
Brain MRI of cortical lesions in cases 1 and 2. FLAIR hyperintensity was seen in the bilateral cerebral cortex in case 1 **(A–E)** and in the left hemispheric cortical region in case 2 **(G–K)** (arrowheads). However, the FLAIR hyperintensities in the cortical regions became shallow and fewer after 3 months in case 1 **(F)** and disappeared after 1 month in case 2 **(L)**.

A previously healthy 20-year-old male patient presented with a witnessed primary generalized tonic-clonic seizure. After about 10 h in a coma, a nervous system examination revealed no limb weakness or sensory symptoms. Poorly marginated, hyperintense lesions in the left frontotemporal parietal lobe and the right frontal lobe were displayed on both FLAIR (Figures 1A, 2A–E) and T2-weighted images of cranial MRI (Figure 1C), which were less evident on T1-weighted (Figure 1B) and diffusion-weighted images (Figure 1D). No epileptic waves were captured on an electroencephalogram (EEG). Laboratory data revealed an increase in the white blood cell count (17.32 × 10^9^/L). A cerebrospinal fluid (CSF) analysis showed a mild increase in leukocytes (80/μl) and protein (48 mg/dl). CSF cytology suggested lymphocytic reaction. CSF culture was negative. Neither immunoglobulin M (IgM) nor IgG of the herpes simplex virus were tested positive in CSF. The patient was diagnosed with viral encephalitis and treated with dexamethasone and acyclovir combined with antiepileptic drugs (sodium valproate) for 2 weeks. He no longer had epileptic seizures and was advised to continue prednisone [40 mg qd (once a day)] orally for 40 days after discharge. The dosage of prednisone was reduced by 5 mg every 5 days until discontinued. However, he had to be readmitted several times within 4 months because of frequent epileptic seizures and headaches with or without a fever. During this period, seizures occurred in two main forms: primary generalized and focal. He received symptomatic treatment each time. A follow-up brain MRI after 3 months suggested that FLAIR hyperintensities in the bilateral cortical regions were shallow and fewer (Figures 1E, 2F). He was readmitted because of dizziness and unsteady gait 3 years later, and a nervous system examination revealed poor left-side ataxia. Cranial MRI revealed FLAIR-hyperintense (Figure 1F), T1-hyperintense (Figure 1G), T2-hyperintense (Figure 1H), and diffusion-hyperintense (Figure 1I) lesions in the pons and left brachium pontis. CSF analysis showed four leukocytes and a mildly increased protein level (51 mg/dl). AQP4-IgG and oligoclonal bands in serum and CSF were negative. The myelin basic protein (MBP) level in the serum and CSF was not elevated. Antibodies of autoimmune encephalitis in the serum and CSF were negative. The cell-based assays (CBAs) revealed that MOG-IgG was positive with titers of 1:10 in the serum. Thus, the diagnosis was corrected to MOG-IgG-associated encephalitis. He received intravenous pulse methylprednisolone (500 mg/day for 3 days), followed by oral prednisone (60 mg qd, with a tapering dose of 5 mg every 1 week) and mycophenolate mofetil [0.75 g bid (twice a day)]. The FLAIR hyperintensities in the brainstem were shallow and smaller after 1 month (Figure 1J). The dosage of prednisone was gradually reduced to 10 mg daily and on maintenance for 1 year. He fully recovered, and MOG-IgG in his serum turned negative after 18 months. No recurrent events occurred during a 28-month follow-up.

### Case 2

A 20-year-old male patient without any previous medical history was admitted to the hospital with twice primary generalized tonic-clonic seizures in 48 h. The neurological examination was normal. The brain MRI showed FLAIR-hyperintense lesions in the left frontal lobe (Figures 2G–K). EEG captured 2- to 3-Hz complex waves of sharp slow frequencies in the right frontal and bilateral occipitotemporal lobes. The laboratory data revealed a normal white blood cell count and blood biochemistry results. The CSF study showed a mild increase in leukocytes (31/μl) and a normal protein level. The antibodies of autoimmune encephalitis and CNS demyelinating disease in the serum and CSF were tested, which indicated non-elevated MBP levels and negative oligoclonal bands and AQP4-IgG. However, a CBA revealed anti-MOG-IgG in his serum (1:100). He was diagnosed with MOG-IgG-associated encephalitis and treated with antiepileptic drugs (levetiracetam) and intravenous pulse methylprednisolone (500 mg/day for 3 days), followed by oral prednisone (60 mg qd) and mycophenolate mofetil (0.5 g bid) induction. The FLAIR hyperintensities in the unilateral cortical regions disappeared after 1 month (Figure 2L). With a tapering dose of 5 mg prednisone every 1 week and low-dose prednisone (10 mg daily) for 6 months, the patient did not experience any relapse during the 16-month follow-up.

### Case 3

**Figure 3:**
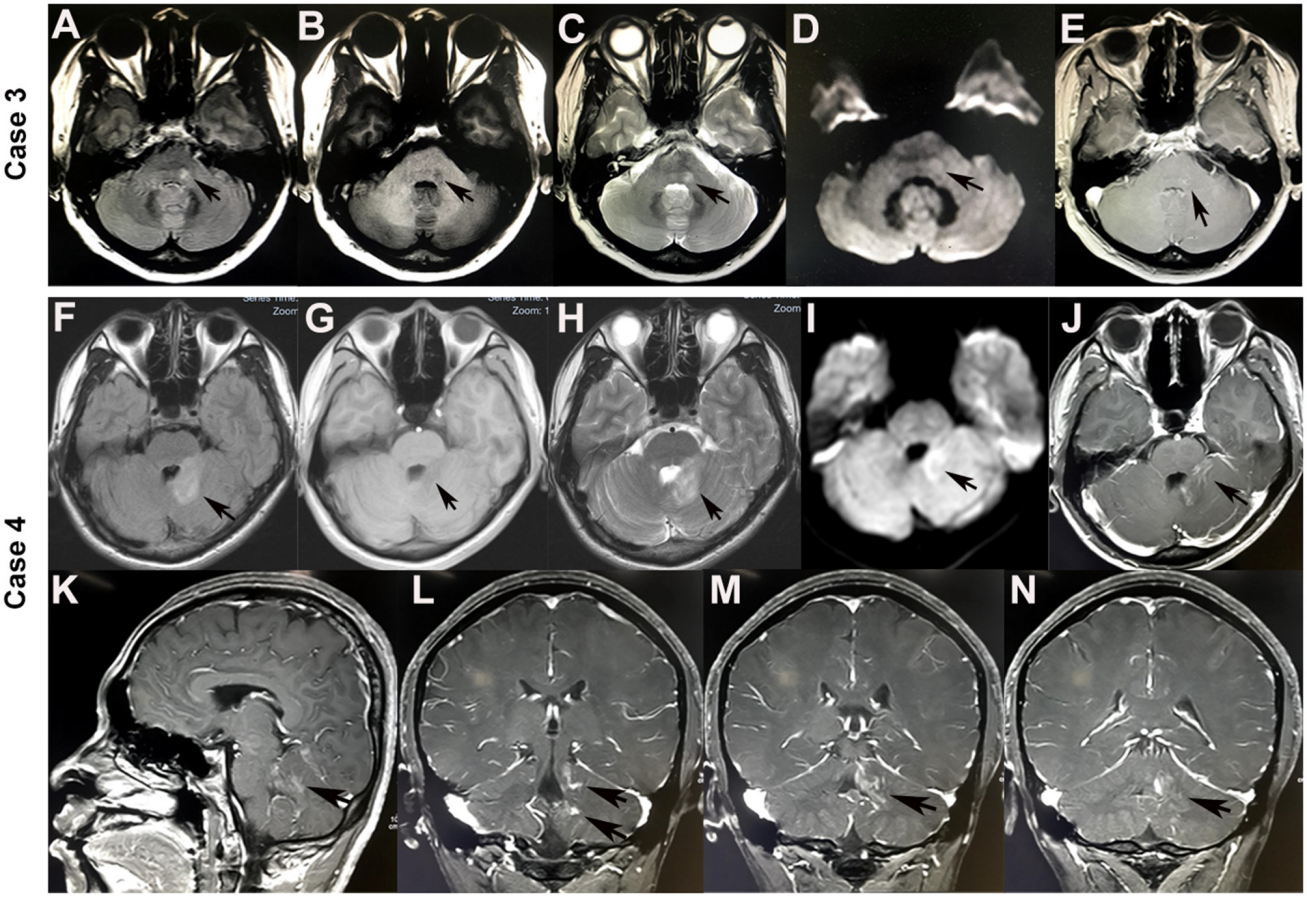
Brain MRI of brainstem lesions in cases 3 and 4. A nodular hyperintense lesion in the left brachium pontis on FLAIR **(A)**, T2-weighted **(C)**, and diffusion-weighted **(D)** images (arrowheads). However, it was hypointense on the T1-weighted image **(B)**, and with a gadolinium-enhanced edge on the T1-weighted image **(E)** (arrowheads). Hyperintense lesions in the left brachium pontis, left cerebellar hemisphere, and cerebellar vermis on FLAIR **(F)**, T2-weighted **(H)**, and diffusion-weighted **(I)** images (arrowheads). However, it was hypointense on the T1-weighted image **(G)**, and with curvilinear post-gadolinium contrast enhancement on the T1-weighted image: axial **(J)**, sagittal **(K)**, and coronal **(L–N)** (arrowheads).

A 51-year-old female patient was admitted to the hospital for left-side facial paralysis and left perioral numbness. Her medical history showed hysterectomy because of uterine fibroids for 15 years. A neurological examination on admission showed left-side peripheral facial paralysis and decreased touch and pain sensation in the left perioral area. Cranial MRI disclosed nodular FLAIR-hyperintense (Figure 3A), T1-hypointense (Figure 3B), T2-hyperintense (Figure 3C), and diffusion-hyperintense (Figure 3D) lesions in the left brachium pontis, with an enhanced edge on T1-weighted images (Figure 3E). No supratentorial lesion was found. The conventional laboratory tests of blood and serum antineutrophil cytoplasmic antibody and anti-myeloperoxidase antibody were negative. The CSF examination revealed a normal cell count (3/μl) and protein level (31 mg/dl). The tests for oligoclonal bands and AQP4-IgG in both blood and CSF cultures were negative. MBP in the serum and CSF was not elevated. However, a CBA revealed MOG-IgG in her serum (1:10). She was diagnosed with MOG-IgG-associated brainstem encephalitis. After a treatment with intravenous pulse methylprednisolone (500 mg/day for 3 days) followed by oral prednisone (60 mg qd, reduced by 5 mg per week) and mycophenolate mofetil (0.5 g bid), the symptoms gradually improved. The patient was in a stable condition during a 6-month follow-up with 10 mg oral prednisone and 1 g mycophenolate mofetil daily.

### Case 4

A 22-year-old male patient presented with dizziness and an unsteady gait. He was admitted to a local hospital and received symptomatic treatment for several days. The symptoms persisted and progressively worsened. He felt weakness in the left-side limbs and was transferred to our hospital. A nervous system examination discovered mild left-side hemiparesis. Cranial MRI revealed T1-hypointense (Figure 3G), T2-hyperintense (Figure 3H), FLAIR-hyperintense (Figure 3F), and diffusion-hyperintense (Figure 3I) lesions with curvilinear post-gadolinium contrast enhancement (Figures 3J–N) in the left brachium pontis, left cerebellar hemisphere, and cerebellar vermis. The CSF analysis showed a mild increase in leukocytes (55/μl) and normal protein levels. Oligoclonal bands and AQP4-IgG were negative in both blood and CSF. MBP in the serum and CSF was not elevated. However, CBAs detected positive anti-MOG-IgG in CSF (1:1) but negative in serum. Considering the limited reliability of anti-MOG-IgG in CSF and his special image presentation, the patient was diagnosed with probable chronic lymphocytic inflammation with pontine perivascular enhancement responsive to steroids (CLIPPERS) syndrome and received the treatment of high-dose intravenous methylprednisolone (500 mg/day for 3 days), followed by oral prednisone starting at 60 mg daily with a tapering dose of 5 mg every 1 week. His symptoms of dizziness and hemiplegia greatly improved, and he was discharged after 20 days of treatment. After 5 months, when the prednisone was reduced to 5 mg/day, the patient developed a sudden primary generalized tonic-clonic seizure and was readmitted to our hospital. Neither nervous system examination nor cranial MRI revealed any abnormality. Slow waves were seen in the left prefrontal, frontal, and temporal regions in EEG. Although cell counts (2/μL) and biochemistry of CSF were normal this time, the anti-MOG-IgG was positive in both serum (1:32) and CSF (1:1) as detected by CBAs. He was diagnosed with MOG-IgG-associated encephalitis; therefore, azathioprine (50 mg bid) and antiepileptic drugs (levetiracetam 0.5 g bid) were added. The patient remained stable during a 6-month follow-up with 10 mg oral prednisone, 1.0 g levetiracetam, and 100 mg azathioprine daily.

## Discussion

Myelin oligodendrocyte glycoprotein (MOG)-IgG–associated disorder (MOGAD) has a specific therapeutic strategy, making early diagnosis and differentiation crucial for developing an individualized treatment approach. However, diagnosis at the first demyelinating event remains a challenge due to overlapping and fickle clinical features of MOGAD. Among all clinical manifestations, cerebral and brainstem damage were quite rare disease phenotypes (<10%) in both pediatric and adult groups (7, 11, 12). MOG-IgG–positive unilateral or bilateral cortical encephalitis detected as FLAIR-hyperintense cortical lesions (as observed in cases 1 and 2) was first reported by Ogawa et al. (5, 6, 8, 13–17) in 2017 as an emerging phenotype of MOGAD (Table 1). Brainstem encephalitis is another infrequent presentation except for cortical encephalitis among the clinical spectrum of MOGAD (7, 13). Patients with brainstem involvement account for about 30% in MOG-IgG-associated encephalomyelitis cases, and isolated brainstem encephalitis that occurs without ON or myelitis is much rarer, accounting for only 1.8% (18).

**Table 1:** Summary of the case reports about MOG-IgG associated cortical encephalitis.

**Study**	**Number of patients (Male: Female)**	**Mean (median) age of onset**	**Main clinical manifestations**	**Abnormal signal in brain MRI**	**Other antibodies**	**Disease course**
Ogawa et al. (8)	4/ (4:0)	34 (37)	Seizures, ON, encephalophagy	Unilateral cortex	None	2/4 had a relapsing disease
Fujimori et al. (5)	1/ (1:0)	46	Seizures, diziniss, paraparesis	Bilateral cortex	None	relapsing disease
Hamid SHM et al. (6)	5 (3:2)	20(10)	Seizures, ON, encephalophagy	Unilateral cortex	None	5/5 had a relapsing disease
Wang L et al. (13)	18 (10:8)	21.3 (22)	Seizures, ON, encephalophagy	Unilateral cortex	5/18 were NMDAR positive	13/18 had a relapsing disease
Fujimori J et al. (15)	6 (3:3)	34	Fever, headache, seizures, paraparesis, lethargy, memory disturbance	Bilateral cortex ± corpus callosum	No data	No data
Kim KH et al. (16)	2 (1:1)	M44 F52	Fever, aphasia, seizures,	Unilateral cortex	None	Monophase course
Ma GZ et al. (17)	1 (1:0)	39	Fever, headache, seizures	Bilateral cortex	None	relapsing disease
Nie HB et al. (14)	1 (0:1)	19	Fever, headache, seizures	Unilateral cortex	None	Monophase course

Cerebral lesions of MOGAD usually look paler, fewer, and less prominent compared with multiple sclerosis cases and can be observed in both T2-weighted and FLAIR images (8, 19, 20). An important basis for diagnosing MOG-IgG cortical encephalitis is unilateral or bilateral cortical lesions on cranial MRI, which are best depicted on FLAIR images (21). In this study, the lesions in case 1 were slightly hyperintense with a hazy border in the bilateral cortical gray matter on FLAIR (Figures 1A, 2A–E) and T2-weighted images (Figure 1C); the lesions became lighter and fewer after 3 months and were displayed only on the FLAIR image (Figures 1E, 2F). Similar abnormal lesions located in the unilateral cortical gray matter were observed in case 2 only on the FLAIR image at the beginning of the disease (Figures 2G–K) and almost disappeared after a month (Figure 2L). Similar to the cortical lesions observed in cases 1 and 2, FLAIR hyperintensity lesions in the cortex or sulcus are also observed in various pathologic conditions, such as meningitis, leptomeningeal metastasis, acute infarction, subarachnoid hemorrhage, and moyamoya disease (22). Thus, patients with MOG-IgG-positive cortical encephalitis, as in cases 1 and 2 in this study, could have been easily misdiagnosed as other diseases.

Brainstem lesions in MOG-IgG-positive cases usually presented a vague, irregularly bordered focus located in different areas of the brainstem, among which the pons was the most commonly involved and accounted for 84.6%. Other common lesions included medulla oblongata (57.1%), cerebellar peduncles (35.7%), and mesencephalon (14.3%) successively (18). The clinical presentations varied according to the areas of brainstem involvement, including cranial nerve palsy, hemiplegia, intractable hiccup, respiratory disturbance, balance difficulties, vertigo, and ataxia. In the present study, case 1 developed brainstem encephalitis 3 years after cortical encephalitis with lesions located in the pons and left brachium pontis, leading to dizziness and left-side ataxia. Lesions limited to the left brachium pontis in case 3 resulted in left-side peripheral facial paralysis and decreased touch and pain sensation in the left perioral area. Positive MOG-IgG in serum and steroid-responsiveness in these two patients confirmed the diagnosis of MOG-IgG-associated brainstem encephalitis. In case 4, diffused lesions in the left brachium pontis, left cerebellar hemisphere, and cerebellar vermis led to multiple neurological deficits, including vertigo and hemiplegia. Curvilinear enhancement signal and negative MOG-IgG in serum led to a misdiagnosis of probable CLIPPERS syndrome. However, with a relapse manifesting as a seizure 5 months later and positive MOG-IgG in serum and CSF, he was eventually diagnosed with MOG-IgG-associated encephalitis. The classical MRI features of CLIPPERS syndrome were punctate or curvilinear gadolinium-enhancing lesions, which were the most prominent in the pons (23). In case 4, the patchy lesions in the left brachium pontis, left cerebellar hemisphere, and cerebellar vermis did not meet the updated CLIPPERS criteria (23). Thus, the presumed diagnosis of CLIPPERS at the first attack was not appropriate. Despite the similar clinical and imaging features of MOG-IgG-related brainstem encephalitis and CLIPPERS (24–26), the diagnostic criteria of CLIPPERS encouraged careful consideration of other possible explanations (23).

Most MOG-IgG are of extrathecal origin; therefore, the presence of MOG-IgG in serum rather than in CSF is recommended as a specific indicator for the diagnosis of MOGAD (4). However, Aoe et al. (27) found that a few patients with MOGAD tested positive for MOG-IgG only in CSF. Patient 4 initially tested positive for MOG-IgG only in CSF, but the detection of MOG-IgG was positive in both serum and CSF when the disease relapsed 5 months later. Thus, we suggest that MOG-IgG should be tested in both serum and CSF simultaneously if possible and re-tested when necessary. Valuable data on regular monitoring of antibody titers in patients of MOGAD are scarce. Nevertheless, disease activity and treatment status may contribute to MOG-IgG titers in serum. Higher median titers were observed during the acute phase rather than the remission stage. Meanwhile, immunotherapy may lead to lower titers (28). In cases 1 and 3, we observed a low titer (1:10) of MOG-IgG during the acute episode, the exact reasons for this are not clear, but several speculations can be offered: first, the effect of glucocorticoid immunotherapy. Second, there may be another unknown pathogenic antibody at the same time. Third, as reported in the literature, some patients have relatively low antibody titers during acute attacks (29).

Cerebrospinal fluid (CSF) findings in our cases presented as pleocytosis of predominant lymphocytes and a normal or mildly elevated protein level around the time of an attack, consistent with previous studies on MOG-IgG-associated encephalitis (5, 6, 8, 13). In an international multicenter study, high MBP levels in the CSF were observed in the MOG-IgG–positive cases, suggesting acute myelin damage during attacks (30). However, the CSF MBP levels were non-elevated in our cases, as observed in patients with MOG-IgG-associated cortical encephalitis in several previous case reports (8, 15, 31). Thus, whether MOG-IgG is directly associated with cortical encephalitis or increased MBP in CSF should be a prerequisite for the diagnosis of MOGAD remains controversial. Notably, positive anti-N-methyl-D-aspartate (NMDA) receptor antibodies were detected in 5/18 of patients in a cohort of MOG-IgG-associated encephalitis (13). This suggested that immune attacks might involve NMDA receptors at the same time. However, antibodies of autoimmune encephalitis in serum and CSF were tested and no sufficient evidence was found that other associated autoimmune antibodies were responsible for the cortical encephalitis and seizures in cases 1 and 2, as observed in many case series/reports (5, 6, 8, 14, 16, 17). The cortical damage in these patients was likely triggered by an episode of demyelination caused by MOG-IgG. Another possibility also existed that an unknown auto-antibody might be involved in disease pathogenesis.

At present, although the long-term prognosis of MOG-IgG-associated encephalitis is not fully understood, the use of corticosteroids and/or plasma exchange or intravenous immunoglobulin remains the current standard treatment in the acute phase (32). Patients with positive MOG-IgG seem to have milder symptoms of neurological impairment (14) and respond more sensitively to corticosteroids than those with positive AQP4 antibodies (33). However, if appropriate treatments are not given in the early course, the symptoms can gradually deteriorate in some MOG-IgG-positive patients (30). Simultaneously, the tapering of oral prednisone slowly following intravenous methylprednisolone is critical on account of a tendency to relapse for rapid withdrawal (28, 34). Long-term immunosuppressive therapy is recommended for patients with recurrence or high risk of recurrence, and mycophenolate mofetil and rituximab are the most commonly used drugs (6). In case 1, the patient did not receive a correct diagnosis and standardized therapy due to limitations in the disease perception and antibody detection methods at the time of the first attack. He also experienced a rapid dose reduction of oral prednisone, leaving intermittent seizures after discharge, and subsequently developed brainstem encephalitis after 3 years. In case 4, the patient was misdiagnosed for the uncertain significance of MOG-IgG in CSF. However, with slow oral prednisone withdrawal, he experienced a relapse representing seizure after 5 months without immunosuppressive agents. Upon experiencing the relapse, cases 1 and 4 were started on mycophenolate mofetil/azathioprine, and they experienced no more events for 28 and 6 months of follow-up, respectively. On the contrary, cases 2 and 3 were diagnosed correctly immediately after the initial attack and recovered completely after the standardized treatment. With the slow oral prednisone and initiation of mycophenolate mofetil, case 2 was stable during 16 months of the follow-up and case 3 remained in clinical remission after 6 months of treatment.

## Conclusion

In summary, this study reported four adult patients who suffered from cortical encephalitis with epileptic seizures and/or brainstem encephalitis during the course of the disease, all of whom were MOG-IgG positive. The four cases might enhance the understanding of MOG-IgG-related disorders. Although MOG-IgG-associated cortical/brainstem encephalitis occurs infrequently, it should be taken into consideration in patients who experience sudden seizure or symptoms of brainstem damage and when MRI reveals cerebral cortical or brainstem lesions. Currently, research on MOGAD is limited and requires further exploration.

## Data Availability Statement

The original contributions presented in the study are included in the article/supplementary material, further inquiries can be directed to the corresponding author/s.

## Ethics Statement

Written informed consent was obtained from the individual(s) for the publication of any potentially identifiable images or data included in this article.

## Author Contributions

All authors listed have made a substantial, direct, and intellectual contribution to the work and approved it for publication.

## Funding

This study was supported by the Projects in Science and Technique Plans of Xingtai City (Grant Number 2021ZC152).

## Conflict of Interest

The authors declare that the research was conducted in the absence of any commercial or financial relationships that could be construed as a potential conflict of interest.

## Publisher's Note

All claims expressed in this article are solely those of the authors and do not necessarily represent those of their affiliated organizations, or those of the publisher, the editors and the reviewers. Any product that may be evaluated in this article, or claim that may be made by its manufacturer, is not guaranteed or endorsed by the publisher.
